# Impact of CircRNAs on Ischemic Stroke

**DOI:** 10.14336/AD.2021.1113

**Published:** 2022-04-01

**Authors:** Miaomiao Liu, Xiaolin Liu, Maorong Zhou, Shao Guo, Kai Sun

**Affiliations:** ^1^Department of Radiology, The Third People’s Hospital of Long Gang District, Shenzhen, China.; ^2^Graduate School of Baotou Medical College, Inner Mongolia University of Science and Technology, Baotou, Inner Mongolia, China.; ^3^Department of Radiology, Baotou Central Hospital, Baotou, Inner Mongolia, China.

**Keywords:** non-coding RNA, Circular RNA, stroke, biomarkers, cerebral ischemia

## Abstract

Circular RNA (circRNA) is a recently discovered class of endogenous non-coding RNA that is generated by cyclization, driven by intron pairing, and regulated by related regulators. An important biological function of CircRNA is acting as a molecular sponge to significantly alter miRNA levels over a short period. Several studies have shown that circRNA is closely related to stroke. Therefore, a better understanding of CircRNA function and regulatory mechanism in ischemic brain will help us for the early detection, early diagnosis, and early treatment of stroke. Here, we summary the biological characteristics, expression of circRNA, and its effect on outcome after ischemic stroke.

Stroke is caused by acute cerebral circulation dysfunction or acute cerebrovascular disease and can be divided into hemorrhagic and ischemic stroke (IS). Among them, IS was the most common, accounting for about 85% of all strokes. Stroke begins with a decrease in brain blood volume, oxygen, and nutrients, which leads to a dynamic progression of blood-brain barrier damage, immune response, inflammatory reaction, oxidative stress, apoptosis, autophagy, neurovascular endothelial damage, and remodeling.

Stroke has become the leading cause of death and disability in China. Its treatment cost accounts for 4.13% of the total national medical expenses and has a high medical, economic, and social burden on patients, their families, and society [1-2]. The therapies most widely used for IS are intravenous thrombolysis and mechanical thrombectomy. However, such treatments are often limited by a narrow time window, potential bleeding risks, limited eligibility criteria, and heavy financial burden for patients and society. Moreover, the pertinence of screening and intervention measures for stroke is insufficient, requiring powerful and effective screening tools. Hence, there is an urgent need to new treatment for stroke. It has been demonstrated that circular RNAs (circRNAs) are tissue-organ specific and enriched in the nervous system [3]. Moreover, it may affect axon growth, neuronal migration and interact with human RNA binding proteins through competitive binding microRNA (miRNA) and participate in brain development and pathogenesis of neurological diseases, muscle diseases, and cerebrovascular diseases. CircRNAs play multiple roles in the pathophysiology of IS by participating in ischemic brain injury, protection of the blood-brain barrier, inhibition of apoptosis, and neuroinflammation [4]. We summarized the biological function of circRNAs and its relationship with stroke. Understanding such fundamental aspects of circRNA can help biological and medical researchers to develop a feasible and accurate screening approach for early-stage stroke to mitigate the risk and reduce the cost of long-term treatment.

## 1. Overview of CircRNA

### 1.1 Characteristics of circRNA

CircRNAs, a special class of endogenous non-coding RNA, are usually *in vitro* and *in vivo* produced by exon or intron cyclization splicing or reverse splicing. According to the genomic origin, CircRNA can be divided into three categories: annular intron RNA (ciRNA), exon RNA (eCircRNA), and exon-intron CircRNA (eiCircRNA). Notably, based on the positional relationship between circRNA and adjacent coding RNA, the aforementioned three CircRNAs can be further divided into five subtypes: exon, intron, antisense, justice overlap, and intergenic [5].

Unlike the structure of linear RNA or other types of RNA, circRNA has a closed covalent ring structure endowing the verified circRNA with several characteristics [6]. CircRNA is less sensitive to exonuclease and more stable than linear RNA because of the structural advantage of lacking free 3’ and 5’ ends. Human circRNAs were initially found in the 1990s. At the time, they were considered aberrant splicing products generated by splicing mistakes. Furthermore, there is generally a low amount of circRNA, and standard methods for investigating linear RNA are ineffective for researching circRNAs. Therefore, their function is understudied [7]. CircRNA was previously found in various species, thanks to advancements in biochemical enrichment approaches and total transcriptome sequencing techniques (RNA-seq).

### 1.2 Biological function of circRNAs

### 1.2.1 A single circRNA acts as a miRNA sponge

Theoretically, circRNA binds with miRNA competitively like a sponge through base complementarity, which is called “miRNA sponge action” [8]. The connections between circRNA and the binding site of miRNA can affect biological function. It has been found that circRNA-CDR1, ciRS-7, circ-ITCH, circ-SRY can act as miRNA sponges [9]. For example, the mammalian sex-determining gene murine SRY (sex-determining region Y) can encode testicular specific circular transcripts. Cirs-7 is a typical circRNA containing more than 70 miR-7 binding sites, which exists in many tissues, especially brain tissues [9].

### 1.2.2 Interaction with RNA binding protein

The most famous protein that interacts with RNA molecules is RNA binding protein (RBP). CircRNA competitively binds to the substrate-binding site of RBP through the mode of storing and transporting RBP and then regulates RBP activity [10]. CircRNA can operate as a miRNA sponge to suppress miRNA activity, engage in target gene splicing, translate genes into proteins, and interact with RBP. RBP interacts with circRNA and stimulate CircRNA production, while circRNA can control the functional activity of RBP. CircRNA can act as a protein sponge or bait to affect cell function, regulate gene transcription, inhibit cell cycle processes, induce apoptosis and promote cell proliferation and survival [11].

### 1.2.3 Regulating the expression of proteins and genes

As we mentioned earlier, one kind of regulatory circRNA, called exon-intron circRNA (EIciRNA), plays a role in transcriptional regulation. EiCircRNA is a multi-exon circRNA that contains one or more non-spliced inserted introns. Some eiCircRNAs located in the nucleus can promote the transcription of their parent genes by interacting with the spliceosome component U1 small ribonucleoprotein (snRNP) [12]. Previous study has demonstrated that EIciRNA can inhibit parent gene transcription by interacting with host U1 snRNP and RNA Pol II to compete with mRNA and influence protein translation [10]. According to Abdelmohsen et al., circRNA (circPABPN1) and its corresponding mRNA competed in the RBP HuR, affecting protein production [13]. Another study [14] found that circANRIL inhibited rRNA processing and ribosomal function while activating p53 in human vascular endothelial cells and macrophages 60s *via* binding to the ribosomal assembly factor pescadillo homolog 1 (PES1). CircRNAs regulate ribosomes and play a certain role in protein expression. These studies all confirmed that ribosomes regulated by CircRNAs play a certain role in protein expression.

### 1.2.4 Other functions

It is also important to point out that there are many other potential features of circRNA, such as competing with mRNA clipping (e.g., the splicing factor muscle blind, mbl) [10]. In addition, it has been reported that circRNA can also affect cell differentiation, participate in pseudogene formation, the regulation of intercellular signal pathway, and stress response, and consequently further impact the occurrence and development of stroke.

## 2. CircRNA expression profile after IS

Biomarkers are highly important for early detection and follow-up monitoring of IS, and they contribute to a thorough understanding of the disease’s etiology and pathophysiology. MiRNAs and lncRNAs have been proposed as possible stroke biomarkers [15,16]. Furthermore, circRNAs have several functions in the onset and progression of IS and are gaining interest as a possible biomarker. Understanding the differential expression of circRNAs as the disease progresses will provide us new biomarkers for the diagnosis and prognosis of IS. Many studies have shown that the expression of circRNAs in brain and plasma is affected during the occurrence of cerebral infarction and the differential expression of circRNA in cells of oxygen glucose deprivation/re-oxygenation (OGD/R), an in vitro model of IS. Moreover, there are differences in the expression of circRNA in patients with different stroke etiology and in the non-ischemic area of IS.

### 2.1 Differential expression of circRNA in brain tissues after IS

Mehta et al. [17] developed the middle cerebral artery occlusion (MCAO) model in male C57BL/6J mice for the first time. At 6, 12, and 24 h after reperfusion, 1320 differential circRNAs were discovered, of which 283 cases changed at different reperfusion time courses. The qRT-PCR analysis revealed that circ-008018, circ-015350, and circ-016128 were upregulated, whereas circ -11137, circ-001729, and circ-006696 circRNAs were downregulated. Their primary biological and molecular activities include biological regulation, metabolic processes, cellular communication, and proteins, ions, and nucleic acids binding. Liu et al. [18] analyzed the mouse brain tissue microarray after MCAO for 45 min and found that 1027 circRNAs changed significantly in the brain tissue 48 h after reperfusion. By analysis of qRT-PCR, mmu-circRNA-40001, mmu-circRNA-013120, and mmu-circRNA-25329 are differential expressed. It regulates cell proliferation and survival mainly by participating in the regulation of the Rap1 pathway and Hippo pathway. Further analysis shows that these circRNA target genes may be involved in regulating different biological processes. Finally, the interaction network of circRNA-miRNA target genes includes 13 miRNAs and their corresponding genes.

Taken together, the changes of circRNAs were related to the genes associated with brain injury and recovery.

### 2.2 Expression profile of circRNA in the cerebral infarction area

Lu et al. [19] investigated circRNA expression in the blood of transient MCAO model in mice and confirmed the chosen circRNA in patients with acute IS (AIS). The findings revealed that 128, 198, 789 circRNAs were substantially altered 5 min, 3 h, and 24 h after IS, with their targeting genes linked to the Hippo signal pathway, extracellular matrix-receptor interaction, and fatty acid metabolism, respectively. Finally, circBBS2 and circPHKA2 were shown to be differently expressed in the blood of patients with AIS. HTS was used to compare the expression of circRNAs in peripheral blood mononuclear cells (PBMC) from five patients with AIS and five healthy subjects [20]. Compared with the normal control group, 521 circRNAs were differentially expressed, of which 373 circRNAs were upregulated, and 148 circRNAs were downregulated. QRT-PCR confirmed that eight circRNAs contained multiple microRNA binding sites. GO and KEGG analysis showed that abnormal expression of circRNAs was involved in many pathophysiological processes of AIS, especially inflammatory and immune processes. The connection between circRNAs and IS induced by MCAO was addressed by Duan et al. [21]. The findings revealed that 87 of the differentially expressed circRNAs had more than two-fold changes. circRNA. 17737, CircRNA.8828, and CircRNA.14479 were all substantially upregulated, whereas circRNA.1059, circRNA.9967, and circRNA.6952 were all significantly downregulated.

Li et al. [22] analyzed the circRNA expression patterns in three patients with AIS and three normal healthy controls in the Han population in southern China. They found that 2270 circRNAs were expressed differently, of which 659 were upregulated, and 1611 were downregulated. De circRNAs may participate in the pathophysiology of AIS through endocytosis, energy metabolism, apoptosis, the FOXO signaling pathway, platelet activation, neurotrophic factor signaling pathway, and VEGF signaling system. The qRT-PCR results showed that hsa-Circ-0005548 was significantly upregulated, while hsa-circ-0000607 and hsa-circ-0002465 were significantly downregulated. In addition, the area under the curve (AUC) values suggests that hsa-circ-0000607 and hsa-circ-0002465 could be potential biomarkers of AIS, and hsa-Circ-0000607 may play a major role in the occurrence and development of AIS by regulating the mir-337-3p/BCL2 axis. Li et al. [23] explored circRNA expression profiles in five patients with LAA stroke and four controls. They found that 182 circRNAs were elevated and 176 were downregulated in patients with LAA stroke. qRT-PCR verified six differentially expressed circRNAs. These circRNAs are mainly involved in chromatin remodeling, autophagy, platelet activation, and brain precursor cell proliferation. The expression level of hsa-circRNA 0001599 was positively correlated with the National Institutes of Health Stroke Scale score (NIHSS) and infarct volume. The area under the receiver operating characteristic curve was 0.805, and the diagnostic sensitivity and specificity were 64.41% and 89.93%, respectively. There are differences in the expression of some circRNAs in the blood of patients with AIS, which may be useful as a diagnostic biomarker or potential therapeutic target of AIS.

### 2.3 CircRNA is differentially expressed in HT22 cells after OGD/R

Lin et al. [24] discovered that 15 circRNAs altered substantially, with 3 upregulated and 12 downregulated. MMU-circRNA-015947 expression is increased and can interact with microRNA to increase the expression of target genes. It contributes to the progression of ischemia-reperfusion damage by involving in apoptosis, metabolism, and immune-related pathways. This research demonstrates that MMU-circRNA-015947 expression is implicated in the process of cerebral ischemia-reperfusion damage and possible for a new molecular target for therapeutic therapy.

### 2.4 The differencial expression of circRNA in stroke patients with different etiology

Aiora et al. [3] reported 219 differentially expressed circRNAs in patients with atherosclerotic and thrombotic stroke, of which 60 upregulated circRNAs showed more than quadruple expression. When comparing atherosclerosis and undetermined stroke, there were 226 circRNAs differentially expressed, 87 circRNAs upregulated, and 139 circRNAs downregulated, of which only one circRNA expression was more than quadrupled. When comparing thrombotic stroke with undetermined stroke, only 8 circRNAs were upregulated, and 9 circRNAs were downregulated. Differential expression of circRNAs in atherosclerosis and cardiac embolism was verified by qRT-PCR. It was found that only ubiquitin Amur52 ribosomal protein fusion product 1 (UBA52) gene HSA_circRNA_102488, which originated on chromosome 19, had statistically significant changes between different etiological subtypes, and the RBP site of hsa_circRNA_102488 was clustered around AGO2 and FUS protein. Finally, functional analysis showed that differentially expressed circRNAs mainly interacted with stroke-related miRNAs.

### 2.5 Differential expression of circRNA in the non-ischemic area after IS

According to Li et al. [25], a total of 2659 circRNAs altered substantially in the ipsilateral thalamus of adult male C57BL/6J mice in the persistent distal MCAO compared to the control group. Among these, 73 circRNAs altered substantially after stroke. CircRNAs are shown to play key roles in subsequent thalamic degeneration and remodeling following a focal cortical acute infarction, according to GO and KEGG analyses. This is the first study to explore circRNA expression in the non-ischemic region of an IS, implying that circRNAs might be a therapeutic target for decreasing subsequent distal neurodegeneration following stroke.

In summary, these studies show that various circRNAs are differentially expressed after the onset of AIS, suggesting that circRNAs have potential application value in the diagnosis, treatment, and prediction of AIS (Table 1).

**Table 1:** Differential expression of circular RNA in IS.

Disease type	Model	Detection method	Change of expression profile	Specific core CircRNAs	Possible major biological processes and functions	Possible major signaling pathways/pathophysiological processes (target genes)	Refs.
Ischemic stroke	Mouse tMCAO model	Microarray analysis and qRT-PCR	1320 CircRNA differentially expressed (among them, 283 cases changed at least one reperfusion time point (> two times), 16 cases changed at all three reperfusion time points)	Three up (Circ_008018, Circ_015350, Circ_ 016128); three down (Circ_011137, Circ_001729, Circ_006696)	Biological regulation, metabolic processes, cellular communication, and binding to proteins, ions, and nucleic acids	Mitogen-activated protein kinase signal, cell cycle, actin cytoskeleton regulation, and local adhesion	Mehta SL et al. [17]
	Mouse tMCAO model	Microarray analysis and qRT-PCR	1027 CircRNA were differentially expressed, of which 914 were upregulated, and 113 were significantly downregulated	mmu_CircRNA_40001 mmu_CircRNA_013120 mmu_CircRNA_4080	Multiple biological processes, cellular signaling pathways, and protein activity	Ten paths, the first two were Rap1 signal pass and Hippo path	Liu C et al. [18]
	Peripheral blood of 80 AIS patients with different etiologies	Microarray analysis and qRT-PCR	1) Atherosclerotic and thrombotic stroke: 219 differential expressions; 2) Atherosclerosis and undetermined stroke: 87 upregulated and 139 downregulated; 3) Patients with thrombotic stroke and unconfirmed stroke: eight were upregulated, and nine were downregulated	HSA_CircRNA_102488	Differentially expressed CircRNAs interact primarily with a group of stroke-related miRNAs, including fatty acid biosynthesis, lysine degradation, arrhythmogenic right ventricular cardiomyopathy, or hypertrophic cardiomyopathy (HCM)	The RBP sites of hsa_CircRNA_102488 were clustered around AGO2 and FUS proteins	Aiora et al.[3]
	Mouse tMCAO model and peripheral blood of patients with AIS	Microarray analysis and qRT-PCR	There were 128,198,789 CircRNA changes at 5 min, 3 h, and 24 h after IS, respectively	CircBBS2, CircPHKA2	The biological processes related to stroke include immune process, metabolic process, and biological adhesion	Hippo signal Pathway, extracellular Matrix-receptor interaction, and Fatty Acid Metabolism	Lu D et al. [19]
	Permanent distal MCAO model in mice (non-ischemic ipsilateral thalamus)	Microarray analysis and qRT-PCR	2659 CircRNAs were differentially expressed (among them, 73 CircRNAs were significantly changed at seven and 14 days after stroke)	mmu_Circ:chr2:74568941-74573626, mmu_Circ: chr8:8639206-8639489, mmu_Circ:chr18:14633543-14636618	Closely related to inflammation and self-repair, CircRNA plays an important role in secondary thalamic nerve degeneration and remodeling after focal cortical infarction	Metabolic pathway, cancer pathway, PI3K-Akt signal pathway, endocytosis, mitogen-activated protein kinase signal pathway, Ras signal pathway, proteoglycan in cancer, Rap1 signal pathway, local adhesion, axon guidance, and other pathways	Li f et al. [25]
	PBMCs in AIS patients	HTS and qRT-PCR	521 Circ RNA differentially expressed (including 373 upregulated and 148 downregulated)	Four up (hsa_cic:chr1:95609447-95616975, hsa_cic:chr15:55640530-55640923); four down (hsa_cic:chr9:80869752-80879232)	Neuroinflammation and neuroimmunity	The pathways involved include metabolic pathways, mitogen-activated protein kinase signaling pathways, some inflammatory pathways, immune pathways, differentiation, and apoptosis-related signaling pathways	Dong Z et al. [20]
	OGD/R treatment of mouse hippocampal HT22 cells	Microarray analysis and QRT-PCR	Three upregulated and 12 downregulated	mmu-CircRNA-015947	-	Apoptosis, metabolism, and immune-related pathways	Lin SP et al. [24]
	Rat MCAO model (six left hemispheric brain tissues)	HTS and qRT-PCR	40 upregulated and 47 downregulated (four days after MCAO)	Three up (CircRNA.17737,CircRNA.8828,CircRNA.14479); three down (CircRNA.1059,CircRNA.9967, CircRNA.6952)	Nervous system development and endocytosis	Cytoplasmic vesicles, vesicles, synapses, cytoskeletons, cytoplasmic vesicles	Duan X et al. [21]
	Three AIS patients and three normal healthy controls	Microarray analysis and qRT-PCR	2270 CircRNAs were differentially expressed, of which 659 CircRNAs were upregulated, and 1611 were downregulated	hsa_Circ_0005548 significantly upregulated, hsa_Circ_0000607 and hsa_Circ_0002465 decreased significantly, hsa_Circ_0000607/mir-337-3p/BCL2 axis	-	Endocytosis, energy metabolism, apoptosis, FOXO signaling pathway, platelet activation, neurotrophic factor signaling pathway, and VEGF signaling pathway	LiS et al. [22]
	Five LAA stroke patients and four controls	Microarray analysis and qRT-PCR	182 CircRNAs upregulated and 176 downregulated	hsa_CircRNA_0001599	-	Chromatin modification, autophagy, platelet activation, and neural precursor cell proliferation	Li S et al. [23]

## 3. Role of circRNA in IS

CircRNA affects the occurrence and rehabilitation of IS in many ways. The role of circRNA in IS needs to be further elucidated and reviewed. Hence, in this section, the potential function and interaction of circRNAs after IS are summaried with a special focus on ischemic brain injury, protection of the blood-brain barrier, inhibition of apoptosis, neuroprotection, and neuroinflammation.

### 3.1 CircRNAs and atherosclerosis

Atherosclerosis is a chronic inflammatory disease of the vascular wall caused by many factors and is the main pathological cause of cardiovascular disease and stroke. In addition, the main causes of IS are carotid plaque rupture and thrombosis. Therefore, understanding the formation of carotid plaque may be helpful to predict and prevent cardiovascular and cerebrovascular events. We aimed to understand the role of circRNAs in serum exons of patients with stable plaque atherosclerosis (SA) and unstable/fragile plaque atherosclerosis (UA). Wen et al. [26] studied the effect of circRNA on the behavior of human umbilical vein endothelial cells (HUVECs) and the mechanism of plaque instability in AS. They found significant differences in the expression profiles of circRNA in serum exons of SA and UA, indicating that circRNA may play a role in carotid plaque instability. In patients with UA, the expression of circRNA-0006896 was positively correlated with triglycerides, low-density lipoprotein cholesterol (LDL-C), and C-reactive protein levels and negatively correlated with albumin levels. However, in patients with SA, the expression of CircRNA-0006896 was positively correlated with LDL-C. These findings suggest that the circRNA-0006896/miR-1264/DNMT1 axis plays an important role in carotid plaque instability by regulating endothelial cell behavior. In addition, circRNA-0006896 may be a therapeutic target for controlling JNK/STAT3 signal transduction in HUVEC. The phenotypic transformation of vascular smooth muscle cells (VSMC) is very important in the pathogenesis of atherosclerosis. Kang et al. [27] identified CDK6 as a key regulator of atherosclerosis, and CDK6 gene knockout inhibited the proliferation of HASMC and HUASMC cells. In addition, the circHIPK3/miR-637/CDK6 axis plays an important regulatory role in the proliferation and apoptosis of VSMC. We believe that circRNA is helpful to develop a simple and noninvasive early screening tool for vulnerable plaques to achieve early prediction and diagnosis of stroke. For example, serum Circ-284: miR-221 has been used as a biomarker for the diagnosis of carotid plaque rupture and stroke [28].

### 3.2 CircRNAs and stroke

### 3.2.1 Ischemic brain injury

Using a circRNA microarray, it was discovered that the level of circHECTD1 was considerably elevated in ischemic brain tissue after transient MCAO in mice and plasma samples of AIS patients [29]. Mechanistically, circHECTD1 acts as an endogenous MIR142 (microRNA 142) sponge, inhibiting MIR142 activity and therefore inhibiting TIPARP (TCDD inducible poly [ADP-ribose] polymerase) production and astrocyte activation via macroautophagy/autophagy. Circhetd1 suppression decreased the infarct size, relieved brain injury, and improved astrocyte activation in transient MCAO animals. CircHECTD1 and its coupling mechanism could play a role in developing cerebral ischemia, giving substantial evidence to promote circHECTD1 as a novel stroke biomarker and therapeutic target. Wu and colleagues [30] reported an enhanced level of circTLK1 in the brain tissue of focal ischemia-reperfusion mice and in the plasma of patients with AIS. CircTLK1 ablation can reduce the infarct volume, minimize neuronal damage, and ameliorate neurological deficits. Furthermore, circTLK1 acted as an endogenous miR-335-3p sponge, inhibiting miR-335-3p activity and increasing 2,3,7,8-tetrachlorodibenzo-p-dioxin-inducible poly (ADP-ribose) polymerase expression, leading to an aggravation of neuronal damage. These results further confirm that circTLK1 is directly associated with IS, indicating the critical role of circRNA on stroke prognosis. The retina is thought to be an outgrowth of brain tissue. However, it is currently unclear if circRNAs can be utilized as common regulators and diagnostic markers for brain and retinal neurodegeneration. Jiang et al. [31] discovered that silencing cGLIS3 reduced ischemia-induced retinal neurodegeneration and that cGLIS3 regulated neuronal cell damage by acting as a sponge for miR-203 and that its level was controlled by EIF4A3. From the standpoint of circRNA, this discovery provides molecular proof that the retina is a window into the brain. cGLIS3 is a common regulator as well as a diagnostic marker of cerebral and retinal neurodegeneration.

### 3.2.2 Protect the blood-brain barrier and inhibit apoptosis

Bai et al. [32] found that circRNA DLGAP4 acts as an endogenous microRNA-143 sponge to inhibit the activity of miR-143. The level of circDLGAP4 in the plasma of patients with AIS and the mouse stroke model significantly decreased. Moreover, *in vivo* experimental results showed that upregulating the expression of circDLGAP4 could significantly reduce the neurological deficit, infarct size, and blood-brain barrier injury. The *in vitro* results showed that over-expression of circDLGAP4 could reduce the downward trend of protein caused by oxygen OGD/R in mice and reduce the expression of mesenchymal cell markers to inhibit the transformation of endothelial cells into mesenchymal cells and reduce the damage of the blood-brain barrier. Furthermore, Zhao et al. [33] showed that the expression of circ-0072309 was significantly decreased while miR100 was significantly increased in the serum of patients with IS and the ischemic hemisphere of MCAO mice. Furthermore, they showed that miR-100 regulates cell survival and apoptosis by directly binding to mTOR, suggesting that he circ_0072309-miR-100-mTOR regulatory axis could be a potential target for the treatment of IS. More evidence has been found that circ_016719 can be directly targeted to miR-29c and control the expression and function of MAP2k6 associated with apoptosis [34]. As the over-expression of circCCDC9 can protect the blood-brain barrier and inhibit apoptosis *via* inhibiting the Notch1 signal pathway, circCCDC9 is considered another new potential therapeutic target for cerebrovascular protection in the acute phase of IS [35].

### 3.2.3 Neuroprotection

There is a significant drop in circMH1 levels in the plasma and periinfarct cortex of photothrombotic (PT) stroke mice and the plasma of patients with AIS. As circMH1 might promote neural plasticity while inhibiting glial cell activation and peripheral immune cell infiltration, circMH1 therapy may improve functional recovery in mice and monkeys following stroke. CircSCMH1 interacts with the transcription factor MeCP2, enabling the inhibition of MeCP2 target gene transcription. Based on such theoretical findings [36], Wang et al. [37] examined CircHIPK2’s involvement in neural stem cell (NSC) differentiation. *In vitro*, suppressing CircHIPK2 aided NSCs in their differentiation to neurons but had no impact on astrocyte differentiation. After stroke onset, microinjected NSCs may migrate to the ischemic hemisphere *in vivo*. Si-circHIPK2NSCs improved neuronal plasticity in the ischemic brain, provided long term neuroprotection, and decreased functional impairments substantially. Dai et al. [38] discovered that circ-HECTD1 knockdown reduced TRAF3 expression *via* targeting miR-133b, reducing neuronal damage after cerebral ischemia. CircSHOC2 in ischemic preconditioning astrocyte exosome (IPAS-EXOs) effectively inhibits neuronal apoptosis and reduces neuronal damage with regulated autophagy on the miR-7670-3p/SIRT1 axis, which may develop IS treatment strategies [39]. A recent report discovered that hsaCirc0078299 and FXN might be considered as new biomarkers of IS to achieve neuroprotection and brain recovery from stroke [40].

### 3.2.4 Neuroinflammation, outcome and recurrence

For the neuroinflammation investigation, around 170 patients with AIS and 170 non-AIS controlled groups were selected to collect the PBMC and serum [41]. PBMC-Circ-DLGAP4 expression was negatively linked to PBMC-miR-143, NIHSS score, CRP, ESR, TNF-, IL-1, IL-6, IL-8, IL-17, and IL-22. It is also related to inflammation and miR-143 expression in patients with AIS. Circ-DLGAP4 has the potential to be used as a novel biomarker for AIS diagnosis and disease monitoring. Similarly, Peng et al. [42] investigated 160 patients with initial AIS and 160 non-AIS as controls and found that CircRNA HECTD1 expression was strongly associated with higher disease risk, disease severity, inflammation, and AIS recurrence. Xu Liu et al. [43] considered circ-STAT3 (signal transducer and activator of transcription) rs2293152 GG as an independent risk factor for stroke recovery. Subgroup analysis showed that the negative effects of rs2293152 GG genotype were higher in females, the elderly, and people with a history of hypertension. In addition, circRNA polymorphism was not associated with IS recurrence. The results show that circ-STAT3 may be a new biomarker to predict the functional outcome after stroke and is also an important factor in the recovery of IS. According to Chen et al., the expression of hsa-circ-0141720 in the serum of patients with ACI increased the most [44]. Further studies showed that the high expression of hsa-circ-0141720 was closely related to the NIHSS score, infarct volume, serum interleukin-6 (IL-6), and plasma C-reactive protein (hs-CRP). The high expression of hsa-circ-0141720 in the serum of patients with ACI is related to the severity of the disease, which can be used as a new serological index in the diagnosis of ACI.

### 3.2.5 Application of circRNAs in other aspects of IS

Identifying individuals at high risk of stroke-related infection (SAI) for preventive antibiotic treatment is essential for patients with AIS. Therefore, to determine whether circFUNDC1 may be a potential predictor of SAI, Zuo et al. [45] investigated 68 patients, of which 26 were infected and 42 were not. Compared to uninfected AIS patients, the level of circFUNDC1 in SAI patients was significantly elevated. The receiver characteristic (ROC) curve demonstrated the predictive significance of circFUNDC1. Furthermore, the level of circFUNDC1 and the number of neutrophils were shown to be positively correlated. In patients with SAI, the ratio of white blood cells to neutrophils was substantially greater than in non-SAI patients. Therefore, circFUNDC1 may be utilized to build a risk prediction model for SAI (Table 2)

For the first time, Xiao et al. [46] demonstrated the comprehensive expression of exosomal circRNAs, showing their potential diagnostic and biological significance in LAA stroke. In peripheral exosomes, RNA-Seq revealed a total of 462 circRNAs, with 25 DE circRNAs. CircRNA competitive endogenous RNA (ceRNA) network and translatable analyses further showed the possible roles of exosomal circRNAs in LAA development. qRT-PCR confirmed two ceRNA pathways involving 5 circRNAs, 2 miRNAs, and 3 mRNAs. ROC curve analysis in the validation cohort indicated two circRNAs as potential new biomarkers, and a logistic model combining two and four circRNAs improved the AUC compared to the individual circRNAs.

**Table 2:** Roles of CircRNA in IS.

Model and organization	Specific genes and their expression changes	Possible signal axis	Major pathophysiological processes that may be involved	Refs.
Ischemic brain tissue of the tMCAO mouse stroke model and plasma of patients with AIS	CircHECTD1 increased significantly	_	Inhibition of circhetd1 expression significantly reduces infarct size, reduces neuronal injury, and improves astrocyte activation	Bing Han et al. [29]
Brain tissue of the mouse model of focal ischemia-reperfusion and plasma of patients with AIS	CircTLK1 increased significantly	_	Knockout of CircTLK1 can significantly reduce infarct volume, reduce neuronal injury and improve neurological impairment	Wu F, et al. [30]
The plasma of patients with AIS and stroke model in mice	CircDLGAP4 decreased significantly	CircDLGAP4/microRNA-143/E6-AP C-terminal domain E3 ubiquitin-protein ligase one axis	Up-regulation of circDLGAP4 expression can significantly reduce neurological impairment, infarct size, and blood-brain barrier damage	Bai Y et al. [32]
IS and LIFR humanized mouse MCAO	Circ-0072309 decreased significantly	Circ-0072309/miR-100/mTOR axis	Regulation of cell survival and apoptosis	Zhao Y et al. [33]
HT22 cells in a mouse model of tMCAO ischemia/reperfusion injury	Circ_016719 increased significantly	Circ_016719/miR-29c/MAP2k6 axis	The 45Circ_016719 gene knockout significantly increased cell proliferation, downregulated apoptosis, and significantly inhibited autophagy	ACT et al. [34]
Mouse tMCAO model	CircCCDC9 decreased significantly	CircCCDC9/Notch1 signaling pathway	Overexpressed circCCDC9 protects the blood-brain barrier and inhibits apoptosis	Wu L, et al. [35]
The plasma of patients with AIS, plasma and periinfarct cortex of mice with photothrombotic stroke	CircMH 1 decreased significantly	CircSCMH1/MeCP2 axis	Circscmh1 can enhance the plasticity of neurons, inhibit the activation of glial cells and the infiltration of peripheral immune cells	Yang L et al. [36]
Mouse tMCAO model	Si-CircHIPK2-NSCs	—	Si-Circhipk2 NSCs increase the plasticity of IS neurons, provide lasting neuroprotection, and significantly reduce functional defects	Wang G et al. [37]
OGD induced HT22 cells and the mouse MCAO model	Circ-HECTD1 significantly upregulated	Circ-HECTD1/miR-133b/TRAF3 axis	Circ-hectd1 knockdown can reduce the death of neurons induced by OGD *in vitro*, reduce the volume of cerebral infarction and reduce the apoptosis of neurons in MCAO mice	Dai Q et al. [38]
*In vitro* oxygen glucose deprivation (OGD) based ischemic preconditioning of astrocyte exosomes (IPAs-EXOs)	CircSHOC2 significantly upregulated	CircSHOC2/miR-7670-3p/SIRT1 axis	Circshoc2 inhibits neuronal apoptosis and alleviates neuronal damage	Chen W et al. [39]
Common RNA-SEQ data of human IS focus tissues and controls	hsa-Circ 0078299 and FXN	—	Neuroprotection and brain recovery	Silva P W et al. [40]
There were 170 AIS patients and 170 non-AIS controls	Downregulation of PBMC-circ-DLGAP4	—	Circ-DLGAP4 was negatively correlated with inflammation and miR-143 expression in AIS patients	Zhu X et al. [41]
There were 160 patients with initial AIS and 160 controls	The expression of CircRNA HECTD1 was higher than the control group	—	CircRNA HECTD1 expression is associated with higher disease risk, disease severity, inflammation, and AIS recurrence	Peng X et al. [42]
Serum levels in 24 asymptomatic and 17 symptomatic patients undergoing carotid endarterectomy	MIR-221 and CircR-284	—	Serum Circ-284:MIR-221 may be used as an early warning factor for carotid plaque rupture and stroke	Bazan HA et al. [44]
Of the 68 AIS patients, 26 were infected, and 42 were not	CircFUNDC1	—	CircFUNDC1 can be used to build a risk prediction model for predicting SAI	Zuo L et al. [45]
Ischemic brain tissue of the tMCAO mouse stroke model and plasma of patients with AIS	cGLIS3	cGLIS3/miR-203/EIF4A3 axis	cGLIS3 is a common regulator and diagnostic marker of cerebral neurodegeneration and retinal neurodegeneration	Jiang Q et al. [31]

### 3.3 CircRNAs after ischemia/reperfusion injury

Ischemia/reperfusion (I/R) damage occurs when blood perfusion returns after IS, causing the region of ischemic injury to extend and worsen. Brain tissue is extremely sensitive to I/R damage, and treatment of I/R damage as soon as possible is important to avoid severe consequences associated with neural cell death. CircRNAs have been shown to play a role in stroke and NSC modulation, and in the recovery of brain function. According to Yang B et al., in cerebral infarction, circTTC3 modulates CIR damage and NSCs via the miR-372-3p/TLR4 axis [47]. Furthermore, Zhang et al. [48] demonstrated that over-expression of hsa-Circ-camk4 increased cell death following OGD/R. Circ-camk4 works primarily *via* “sponging” miRNAs. The pathways most strongly involved in circ-camk4 regulation were those acting at glutamatergic synapses, as well as the MAPK signaling pathway, calcium signaling pathway, and apoptosis pathway, all of which are involved in neuronal cell response to I/R injury. These findings suggest that circ-camk4, as a type of circRNA, may play a key role in brain I/R injury.

## 4. Potential role of circRNAs in the treatment of IS

It is important to develop a new strategy for neuroprotection and neural tissue recovery after IS. Natural regulatory peptides can significantly affect brain activity and have high safety without side effects. Preparations generated from natural regulatory peptides have effectively created therapeutic techniques based on neuroprotection, anti-inflammatory, nerve stimulation, and anti-stress activation. The molecular genetic alterations in the brain following cerebral ischemia, as well as the mechanism of polypeptide medicines, are unknown. This restricts the use of neuroprotective peptides and makes developing new and more effective medicines to target brain function challenging. Transcriptome analysis is a potential approach for investigating the molecular mechanisms of cerebral ischemia damage and the neuroprotective effects of peptide medications. In addition to investigating the role of mRNA in protein production, researchers must also investigate the role of regulatory RNA in ischemia when developing new neuroprotective methods. MiRNAs and circRNAs, which are mostly expressed in the brain, are the most intriguing. CircRNA may bind with miRNAs and decrease their activity, preventing miRNA-mediated mRNA from being produced. Understanding the circRNA/miRNA/mRNA system is critical for insight into the process of brain damage and healing. Ischemia-induced gene activity changes and peptide-mediated transcriptome spectrum alterations in experimental ischemia were reviewed by Filipenkov et al. [49], who also explained the underlying concept of peptide regulation in ischemia-induced damage. He et al. [50] successfully established the MCAO rat model for the first time and injected 10 mg/kg fluoxetine hydrochloride intraperitoneally for 14 days to study the function of fluoxetine in cerebral IS and the identification of fluoxetine mediated circRNA-miRNA-mRNA axis. After that, triphenyl ammonium tetrachloride staining was used to determine the location of the cerebral infarction. Their findings indicated that fluoxetine might ameliorate brain damage following IS and that the circMap2k1/miR-135b-5p/Pidd1 axis may be implicated.

## 5. Summary and prospect

One in every six people worldwide will have a stroke. However, the underlying process is still unknown, and current therapeutic options are limited. CircRNA is a novel form of endogenous non-coding RNA with a covalently closed circular structure. CircRNA, a member of the non-coding RNA family, was originally thought to be incapable of encoding due to a lack of 5’caps and 3’polya tails. Recent research has shown that circRNAs may be translated in a cap-independent manner by acting as the sequence of ribosomal entry sites (IRES) inside circRNAs to facilitate the direct binding of initiation factors or ribosomes to translatable circRNA [51]. CircRNAs have been discovered to be significantly expressed in the central nervous system, indicating that they are nerve specific. Many studies have found that ncRNA expression changes considerably following the onset of AIS. CircRNA is strongly connected to stroke severity and inflammatory response and plays a key role in stroke diagnosis, prognosis, and therapy. Indeed, the molecular and cellular processes that occur following an IS are complicated and staggered. Further studies are required on the use of circRNAs as biomarkers for stroke diagnosis and prognosis. With the development of new sequencing technology and bioinformatics methods, many circRNAs have been identified in different organisms and tissues. These findings have expanded people’s understanding of the classification and function of RNAs. The relationship and specific mechanism between CircRNAs and stroke occurrence, development, and prognosis will be gradually clarified. Moreover, the basic principle and molecular mechanism of peptide regulation in ischemia-induced injury will need to be further explored to enable the early detection and intervention of stroke.
